# NBR1-Mediated Selective Autophagy in Plant Development and Stress Responses

**DOI:** 10.3390/plants15091350

**Published:** 2026-04-28

**Authors:** Xinye Li, Yali Duan, Jiyang Zhou, Peifeng Yu

**Affiliations:** Xinjiang Key Laboratory of Biological Resources and Genetics Engineering, College of Life Science and Technology, Xinjiang University, Urumqi 830046, China; lee2186@163.com (X.L.); 15009091508@163.com (Y.D.)

**Keywords:** NBR1, selective autophagy, stress adaptations, protein degradation

## Abstract

Autophagy is a conserved degradation pathway essential for cellular homeostasis in plants. Selective autophagy confers cargo specificity through receptors, among which *NEIGHBOR OF BRCA1 GENE1* (NBR1) is one of the best-characterized. NBR1 mediates the selective turnover of ubiquitinated or stress-damaged cargoes, including protein aggregates and damaged organelles, by linking them to ATG8-decorated autophagosomes via its AIM and UBA domains. This process supports proteostasis, plant development, and adaptation to abiotic stresses, including heat, drought, chilling, salinity, and heavy metals, as well as biotic stresses from bacteria, fungi, viruses, and oomycetes. In this review, we summarize current advances in understanding NBR1 structure, evolutionary conservation, and cargo recognition mechanisms, and highlight its interplay with phytohormone signaling and the ubiquitin–proteasome system (UPS) in shaping plant growth and stress resilience.

## 1. Introduction

Autophagy, meaning “self-eating”, is a highly conserved cellular degradation and recycling process that plays a crucial role in maintaining cellular homeostasis [1]. Through this membrane trafficking catabolic process, various cytoplasmic damaged or unnecessary components, including misfolded proteins, protein aggregates, and defective organelles, are selectively or non-selectively captured and transported to the vacuole/lysosome, where they are broken down into smaller molecules for recycling [2,3,4,5,6]. This process not only removes harmful cellular materials but also recovers nutrients and building blocks, thereby contributing to cellular quality control and metabolic balance [4,6].

**Figure 1:**
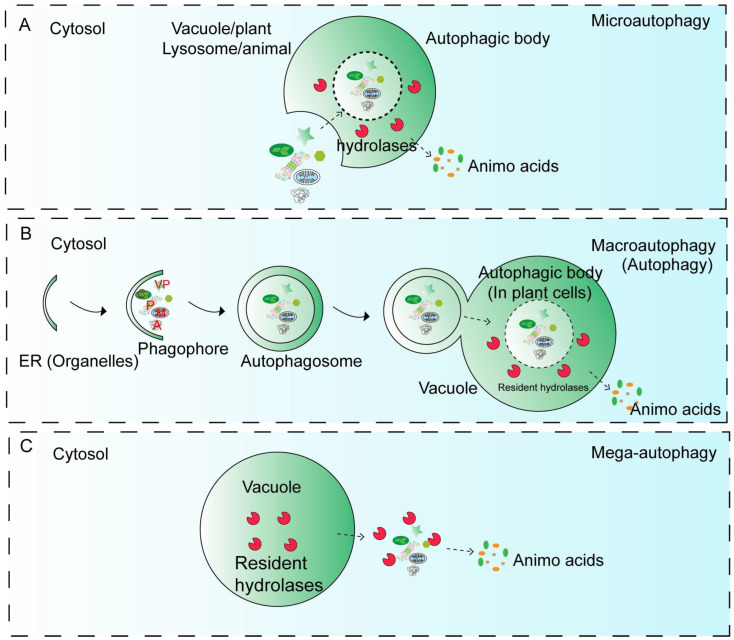
Morphological Steps of Main Types of Autophagy in Plants. Plant cells exhibit three main types of autophagy: microautophagy, macroautophagy, and mega-autophagy. (**A**) In microautophagy, cytosolic components are directly sequestered by invagination, protrusion, or septation of the lysosomal/vacuolar limiting membrane. This process leads to the internalization of cargo into the organelle lumen as vesicular structures (autophagic bodies), without the formation of a separate double-membraned autophagosome. The internalized material is degraded by resident hydrolases, and the resulting amino acids and other metabolites are released for cellular reuse. (**B**) Macroautophagy involves the enclosure of cellular components destined for degradation within double-membraned vesicles known as autophagosomes, which fuse with vacuoles in plant cells, where their contents are exposed to hydrolytic enzymes for breakdown. (**C**) Mega-autophagy is a large-scale degradative process associated with vacuolar membrane permeabilization or rupture, releasing hydrolases that degrade cytoplasmic components, often during developmental remodeling or programmed cell death. Abbreviations: A, protein aggregates; C, chloroplast; M, mitochondrion; P, 26S-proteasome; VP, vacuole protein.

Based on the mechanisms used to deliver cellular materials to the vacuole for degradation, plant autophagy is classified into three main types: microautophagy, macroautophagy, and mega-autophagy [6,7] (Figure 1A–C). In microautophagy, the tonoplast directly invaginates to sequester cytoplasmic components at the vacuolar surface, followed by membrane scission that releases the encapsulated material into the vacuolar lumen as autophagic bodies (Figure 1A).

Conversely, macroautophagy initiates with a cup-shaped, double-membrane phagophore that expands to sequester cytoplasmic cargo before sealing into a mature autophagosome (Figure 1B). Mega-autophagy, an extreme plant defense mechanism exclusive to plants, triggers programmed cell death during pathogen invasion by permeabilizing the tonoplast to release vacuolar hydrolases that degrade cytoplasmic material in situ [7,8] (Figure 1C). In animals, chaperone-mediated autophagy (CMA) provides a vesicle-independent route where the chaperone Hsc70 recognizes a specific KFERQ-like motif on target proteins to facilitate their direct transport into vacuoles via LAMP-2A; however, plants appear to lack both this specialized pathway and its associated genetic machinery.

Over the past several decades, macroautophagy (hereafter termed autophagy) has been the most widely recognized and extensively studied form of autophagy in plants [6,9]. Within this pathway, cargo selectivity is largely conferred by selective autophagy receptors, among which NBR1 has emerged as one of the best-characterized receptors in plants [10,11]. NBR1 acts as a molecular bridge between ubiquitinated cargo and the core autophagic machinery by simultaneously recognizing ubiquitinated substrates via its ubiquitin-associated (UBA) domain and interacting with ATG8 on the phagophore membrane via its ATG8-interacting motif (AIM) [12,13]. Through this adaptor function, NBR1 mediates the selective sequestration of misfolded proteins, stress-induced protein aggregates, and other damaged macromolecular complexes into autophagosomes for subsequent vacuolar degradation.

In this way, NBR1-mediated selective autophagy complements bulk autophagy by conferring substrate specificity, particularly under abiotic and biotic stress conditions that intensify proteotoxic and oxidative damage. Accumulating evidence indicates that plant NBR1 is not merely a cargo receptor, but also a key regulatory node integrating protein quality control, stress signaling, environmental adaptation, and plant growth and development. In this review, we highlight the pivotal role of plant NBR1 as a central hub linking selective autophagy to adaptation to a broad spectrum of abiotic stresses, including extreme temperatures, osmotic imbalance, and heavy metal toxicity, as well as to biotic stresses caused by bacterial, viral, and fungal pathogens. We further discuss the functions of NBR1-mediated selective autophagy in plant growth and development, with particular emphasis on its interplay with the UPS in coordinating plant development and stress responses.

## 2. The Autophagy Mechanism

### 2.1. The Autophagy Pathway

**Figure 2:**
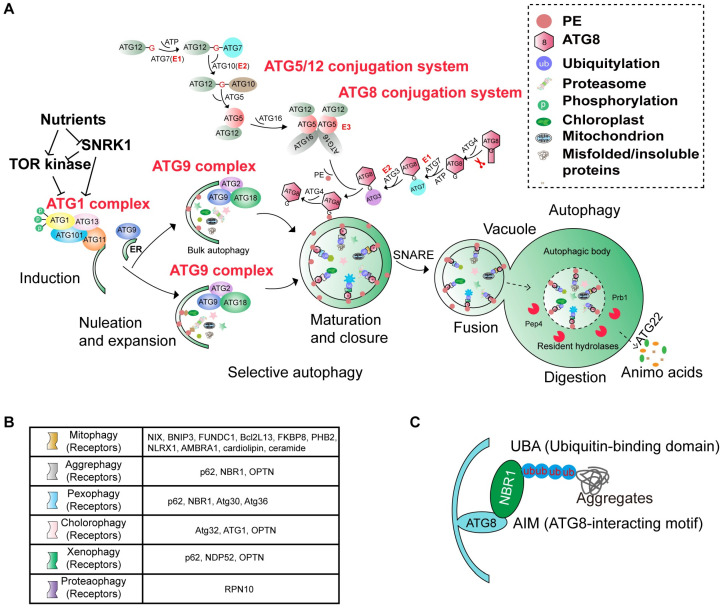
Core Machinery of Autophagy and Selective Autophagy Pathways. (**A**) Autophagy is induced by nutrient signaling through TOR inhibition and SnRK1 activation, leading to activation of the ATG1 complex. Phagophore nucleation and expansion require the ATG9 complex and membrane trafficking. Two ubiquitin-like conjugation systems ATG5-ATG12 and ATG8-PE drive membrane elongation, cargo recruitment, and autophagosome closure. The mature autophagosome fuses with the vacuole, where the inner autophagic body is degraded by resident hydrolases and recycled into amino acids. (**B**) Major forms of selective autophagy are mediated by cargo-specific receptors that link substrates to ATG8, including mitophagy, aggrephagy, pexophagy, chlorophagy, xenophagy, and proteaphagy. (**C**) NBR1-mediated recognition of ubiquitinated protein aggregates via its UBA domain and interaction with ATG8 through the AIM. Abbreviations: Pep4, vacuolar aspartyl aminopeptidase (proteinase A), Prb1, the vacuolar serine protease Proteinase B.

Autophagy initiation is tightly controlled by the target of rapamycin (TOR) complex, a central serine/threonine kinase that acts as a key negative regulator of the pathway [14]. By integrating metabolic status and abiotic stress signals, TOR determines whether cellular conditions are permissive for autophagy induction [6]. Under nutrient-rich and non-stress conditions, active TOR inhibits autophagy through phosphorylation of ATG13, which disrupts its interaction with the ATG1 kinase [15]. Upon TOR inactivation, the ATG1-ATG13 complex assembles and oligomerizes at the phagophore assembly site (PAS), thereby initiating autophagosome biogenesis [16,17] (Figure 2A). In many cases, the PAS is closely associated with the endoplasmic reticulum (ER), where phagophore formation begins, giving rise to the cup-shaped isolation membrane that ultimately expands into the autophagosome [18,19] (Figure 2A).

The biogenesis of plant autophagosomes is mediated by a highly conserved set of approximately 40 autophagy-related (ATG) proteins. These components do not function independently but instead assemble into several core complexes that coordinate phagophore formation. The process is initiated by the ATG1-ATG13 kinase complex [20,21], which regulates the recruitment of ATG9-containing vesicles and associated factors such as ATG18 [22,23,24,25] (Figure 2A). In parallel, the ATG6-ATG14-VPS15-VPS34 phosphatidylinositol 3-kinase (PI3K) complex generates a phosphatidylinositol 3-phosphate (PI3P)-enriched membrane environment that supports phagophore nucleation [26].

Membrane expansion is subsequently driven by two ubiquitin-like conjugation systems. In this process, ATG8 is first cleaved by the cysteine protease ATG4 to expose a C-terminal glycine residue, a prerequisite for its lipidation. This modification enables a cascade involving the E1-like enzyme ATG7, the E2-like enzymes ATG3 and ATG10, and the E3-like ATG5-ATG12-ATG16 complex, leading to the conjugation of ATG8 to phosphatidylethanolamine (PE) and the formation of the ATG5-ATG12 complex [6,26,27,28,29] (Figure 2A).

### 2.2. The NBR1-Mediated Selective Autophagy

Beyond its foundational role in non-selective cytoplasmic recycling, autophagy has evolved into a highly selective catabolic pathway mediated by the recruitment of specialized selective autophagy receptors (SARs) (Figure 2B). These receptors provide molecular specificity by recognizing and tethering distinct cellular cargoes and directing them to the autophagic machinery, where they are subsequently degraded in the vacuole. In plants, this framework supports a range of cargo-specific autophagic pathways, many of which converge on NBR1 as a central adaptor (Figure 2B,C).

Through NBR1, autophagy can selectively target diverse cellular substrates, including aggregated or misfolded proteins via aggrephagy, damaged or excess peroxisomes through pexophagy, and impaired chloroplasts via chlorophagy. NBR1-dependent pathways have also been implicated in immune-related processes such as xenophagy, which restricts intracellular pathogens, as well as proteaphagy, which mediates proteasome turnover and recycling [30,31] (Figure 2B). Collectively, these pathways highlight how NBR1-mediated selectivity expands the functional scope of autophagy in maintaining cellular homeostasis and stress adaptability in plants.

### 2.3. Structure, Evolution, and Function of NBR1

In plants, NBR1 is one of the most prominent effectors of selective autophagy, functioning as a central adaptor for the recognition and delivery of specific cargoes to vacuoles for degradation. NBR1 also plays a key role in plant adaptation to diverse environmental stresses by facilitating the selective autophagic clearance of cytotoxic protein aggregates and damaged cellular components that accumulate under conditions such as heat, drought, salinity, and oxidative stress. Loss of NBR1 function renders plants more sensitive to these stresses, underscoring its essential role in maintaining cellular homeostasis and conferring stress tolerance.

**Figure 3:**
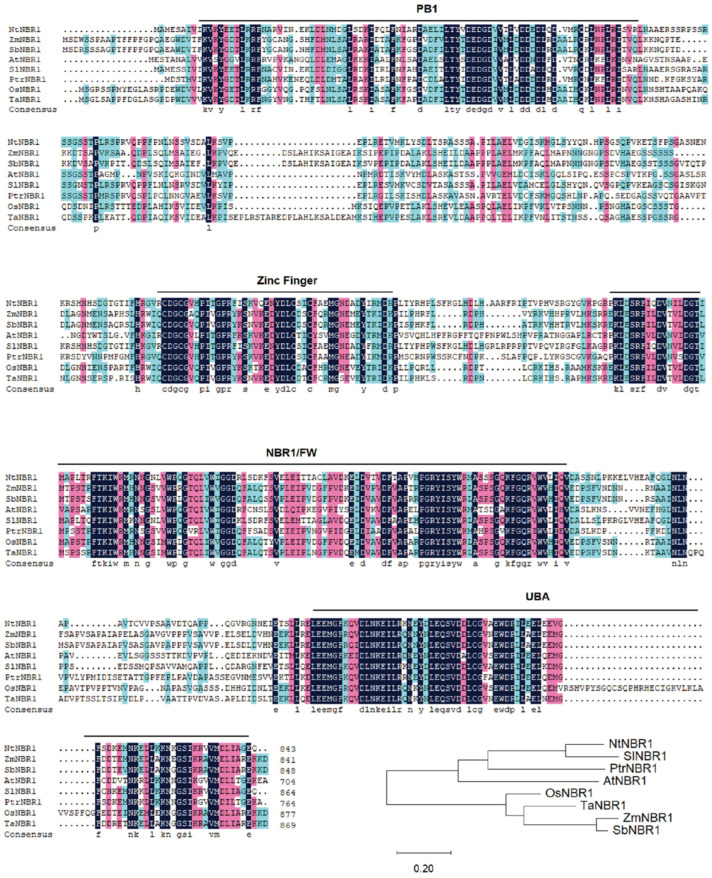
Potein Sequence Alignment and Phylogeny of NBR1 and its Relatives in Other Plant Species. Sequence alignment of NBR1 proteins from *Arabidopsis* and other species was performed using DNAMAN (https://www.dnaman.net/download.html) software. Black and color boxes show identical and similar amino acids, respectively. The PB1, Zinc finger, NBR1/FW, UBA domains, as predicted by PFAM, are indicated in black lines. Species abbreviations: *Arabidopsis thaliana* (At), *Zea mays* (Zm), *Sorghum bicolor* (Sb), *Oryza sativa* (Os), *Populus trichocarpa* (Pt), *Solanum lycopersicum* (Sl), *Nicotiana tabacum* (Nt), *Triticum aestivum* (Ta). Phylogenetic relationship of NBR1 genes with relatives from 8 representative plant species. The tree was generated using a maximum-likelihood method embedded in RAxML [35]. Support for nodes was assessed with 1000 bootstrap replicates. 0.2 serves as the scale bar of the phylogenetic tree, representing the genetic distance unit used in this analysis.

Analogous to mammalian SQSTM1 and p62, plant NBR1 serves as a versatile adaptor that targets aggregated proteins and damaged organelles for selective autophagic degradation [32,33]. The functional versatility is conferred by its modular domain architecture, which enables the receptor to capture, cluster, and deliver diverse substrates to the vacuole. The N-terminal Phox and Bem1 (PB1) domain drives head-to-tail self-oligomerization, an evolutionarily conserved feature shared with p62, thereby promoting the formation of higher-order assemblies that concentrate ubiquitinated cargo and enhance binding avidity (Figure 3). Located in the central region, the ZZ-type zinc finger and FW domains are hallmark structural motifs of the NBR1 family that expand its protein–protein interaction repertoire and may contribute to substrate recognition beyond strictly ubiquitin-dependent mechanisms (Figure 3).

Recruitment to the autophagy pathway is mediated by the LIR/AIM, which directly interacts with ATG8 proteins on the expanding phagophore membrane, thus tethering the NBR1–cargo complex to sites of autophagosome biogenesis (Figure 2C). At the C terminus, plant NBR1 typically contains two UBA domains, of which UBA2 shows strong specificity for K63-linked polyubiquitin chains, a linkage topology frequently associated with selective autophagic degradation [34] (Figure 3). Through the coordinated action of these domains, NBR1 functions as a molecular adaptor that bridges specific cellular cargoes to the core autophagic machinery, thereby facilitating stress-induced proteostatic turnover.

Phylogenetic analysis of plant NBR1 proteins reveals that NBR1 homologs are widely conserved across monocots and dicots. The unrooted tree shows that NBR1 sequences cluster largely according to species lineage, with monocot homologs (e.g., *Zea mays*, *Oryza sativa*, *Triticum aestivum*, *Sorghum bicolor*) forming a distinct clade, separate from dicot homologs (e.g., *Arabidopsis thaliana*, *Nicotiana tabacum*, *Populus trichocarpa*) (Figure 3). Despite sequence divergence, all NBR1 homologs retain the characteristic modular domain architecture, including the PB1, ZZ-type zinc finger, FW, and UBA domains, highlighting the structural conservation of functional motifs critical for selective autophagy across plant species (Figure 3). These observations suggest that NBR1-mediated selective autophagy represents a conserved mechanism for stress adaptation in both monocot and dicot plants.

## 3. NBR1-Dependent Selective Autophagy in Plant Development

Autophagy plays versatile roles in plant growth and development, contributing to the regulation of cell differentiation, vegetative growth, reproduction, senescence, and nutrient remobilization [36,37,38,39]. Over the past decades, extensive studies in *Arabidopsis* and other plant species have revealed that autophagy is broadly involved in multiple developmental processes, particularly in reproductive development, seed filling and maturation, vascular development, and biomass accumulation.

During the vegetative phase, core autophagic components, such as ATG8c, ATG8d, and ATG18d in Populus, alongside ATG2 and ATG8 in *Arabidopsis*, contribute significantly to root and stem development, vascular formation, and overall biomass accumulation [40,41]. As the plant transitions to the reproductive phase, autophagy continues to play pivotal roles. For example, the predominant expression of several *ATGs* (including *AtATG1*, *AtATG5*, *AtATG6*, *AtATG8d*, *AtATG8h*, and *AtATG18e*) in *Arabidopsis* pollen grains underscores their necessity for pollen development and male fertility [38,42,43,44,45]. Ultimately, this autophagic regulation extends to the final stages of the life cycle, where components like ATG12 in maize, ATG5 and ATG6 in barley, and ATG7 in rice are crucial for proper seed maturation and grain filling [46,47,48]. Notably, these ATGs may be regulated by UPS. For example, both ATG5 and ATG7 are degraded by the UPS during seed development, although the specific E3 ligases still remain unknown [49].

In our previous study, we found that both autophagy and the UPS are required for proper seed development, although their contributions appear to be temporally distinct. A coordinated interplay between the UPS and autophagy characterizes seed development. The early developmental stages rely primarily on UPS-mediated degradation; however, as development progresses towards maturation, a functional relay occurs wherein elevated autophagic flux supersedes the declining UPS machinery [49]. Notably, the protein abundance of NBR1 decreased during seed development, and treatment with concanamycin A (ConA), an inhibitor of autophagic flux, stabilized NBR1 protein levels during seed development, indicating that NBR1 is subject to autophagic degradation. However, direct evidence for the interaction between NBR1 and proteasome subunits is still missing [49].

Interestingly, proteasome turnover during early seed development does not appear to depend on RPN10, a well-characterized autophagy receptor involved in proteaphagy [30]. In the *rpn10* mutant, the abundance of proteasome subunits was not stabilized during early seed development, suggesting that their degradation at this stage is unlikely to occur through RPN10-dependent proteaphagy [49]. These observations therefore raise the possibility that an alternative selective autophagy pathway, potentially involving NBR1, contributes to proteasome turnover during early seed development.

**Figure 4:**
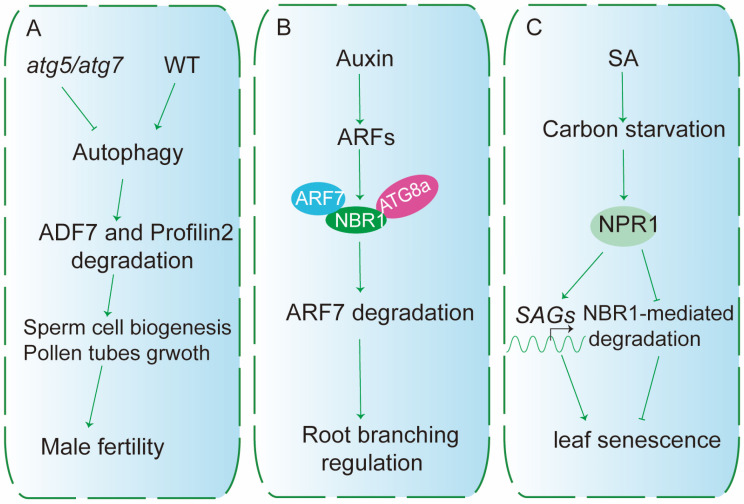
NBR1-mediated Autophagy Regulates Plant Diverse Developmental Processes in Plants. (**A**) Reproductive development. In WT plants, autophagy degrades ADF7 and profilin2, which is required for normal sperm cell biogenesis, pollen tube growth, and male fertility. In *atg5* or *atg7* mutants (lacking core autophagy machinery), this degradation is blocked, leading to fertility defects. (**B**) Auxin-regulated root branching. Auxin signaling induces ARF7, which transcriptionally upregulates NBR1. NBR1 then interacts with ATG8a to initiate selective autophagy of ARF7, modulating root branching. (**C**) Leaf senescence under carbon starvation. Carbon starvation and SA signaling converge on NPR1. NPR1 activates both autophagy (via NBR1-ATG8a) and senescence-associated genes (*SAGs*).

Recent studies have shown that NBR1-mediated selective autophagy also contributes to plant growth and development through the selective removal of cargos involved in these processes [50]. For instance, NBR1-mediated selective autophagy regulates male fertility by promoting the degradation of two actin depolymerization factors, ADF and profilin2. In *atg5* and *atg7* mutants, defective autophagy results in the accumulation of these two factors, thereby impairing male fertility [45] (Figure 4A).

Selective autophagy has been well characterized in plant stress responses, yet its functions during normal development remain comparatively underexplored. Emerging evidence, however, indicates that selective autophagy is also active under physiological conditions. In *Arabidopsis*, the receptor NBR1 mediates autophagic turnover of the Auxin-Response Factor 7 (ARF7) during lateral root initiation, through the regulation of prebranch site (PBS) specification [51] (Figure 4B). Similarly, the ER-phagy receptor AtSec62 is indispensable for normal vegetative growth and pollen development [52]. Consistent with these observations, plants sustain a basal level of autophagic activity during routine growth, facilitating the removal of misfolded proteins, damaged organelles, and metabolic by-products to maintain cellular homeostasis [53]. NBR1, which recognizes ubiquitinated protein aggregates, is constitutively expressed and likely contributes to this housekeeping function. Moreover, Salicylic acid (SA) has been reported to induce carbon starvation-associated responses by inhibiting autophagy in an NPR1-dependent manner, which consequently accelerates NBR1 degradation [54] (Figure 4C).

## 4. NBR1-Dependent Selective Autophagy in Abiotic Stresses

Abiotic stresses severely impair plant growth and productivity by disrupting proteostasis and redox homeostasis. Environmental stresses such as drought, salinity, extreme temperatures, and heavy metal exposure trigger the excessive accumulation of ROS, which not only inflict oxidative damage on cellular components but also promote protein misfolding and aggregation. To cope with these adverse conditions, plants have evolved sophisticated antioxidant enzyme systems and phytohormone-mediated signaling networks that help maintain cellular homeostasis and enhance stress tolerance. Moreover, NBR1-mediated selective autophagy has emerged as an important adaptive mechanism in plant stress responses, contributing to the clearance of unwanted proteins, toxic aggregates, and damaged organelles.

### 4.1. NBR1-Mediated Selective Autophagy in Plant Drought and Heat Stress Tolerance

NBR1-mediated autophagy serves as a central cytoplasmic quality control pathway that helps plants maintain proteome integrity and cellular homeostasis under abiotic stress. Among these stresses, heat and drought pose major threats to cellular proteostasis. In particular, heat stress promotes widespread protein misfolding and structural damage, leading to the accumulation of insoluble protein aggregates that compromise proteome integrity [12]. These aberrant protein assemblies can induce *NBR1* expression, which in turn contributes to the upregulation of heat shock protein (*HSP*)-responsive genes. By recognizing ubiquitinated cargo and interacting with ATG8 on the phagophore membrane, NBR1 directs aggregated proteins to autophagosomes for vacuolar degradation, thereby mitigating proteotoxic stress.

**Figure 5:**
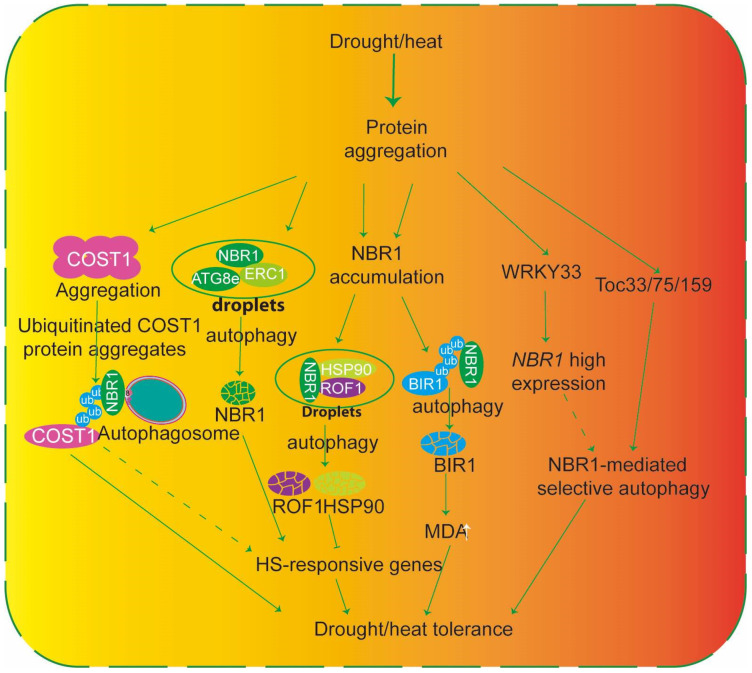
Drought and heat stress promote protein misfolding and aggregation, leading to the accumulation of ubiquitinated protein aggregates (e.g., ubiquitinated COST1). These aggregates are recognized by the cargo receptor NBR1, which interacts with ATG8e to deliver them into autophagosomes for degradation. NBR1 expression is induced under stress, and its accumulation enhances autophagic flux. The resulting clearance of toxic aggregates reduces MDA levels, upregulates heat-shock-responsive genes, and improves drought/heat tolerance. Additional regulatory components (ERC1, ROF1, HSP90, BIR1, WRKY33, and Toc33/75/159) as well as NBR1 droplet formation are depicted in the diagram. Solid arrows indicate experimentally supported interactions, while dashed arrows represent proposed or indirect relationships.

Reverse genetics analyses have significantly advanced our understanding of how NBR1-mediated selective autophagy mitigates proteotoxic stress triggered by heat and drought. In tomato, silencing *NBR1* homologs impaired heat tolerance by preventing the clearance of heat-induced protein aggregates. Mechanistic studies further showed that an aggregation-prone variant of maize FLOURY2 (FL2) lacking its N-terminal signal peptide (FL2SP), when expressed as an FL2SP-GFP fusion in the *nbr1* mutant background, is a substrate for NBR1-dependent autophagic degradation [55]. Similarly, in *Arabidopsis*, the constitutively stressed protein COST1 aggregates under drought conditions and interacts with NBR1, thereby promoting selective autophagy and ATG8 recruitment to alleviate proteotoxic stress [56] (Figure 5).

Beyond the cytosolic proteome, NBR1 also mediates the selective clearance of damaged organelles. Independent studies have revealed that photodamaged chloroplasts and components of the chloroplast outer envelope membrane (TOC) (Toc33, Toc75, and Toc159) are targeted for NBR1-dependent autophagic degradation in response to environmental cues [10,57], a process facilitated by K63-linked polyubiquitination, which marks organellar components for recognition and vacuolar delivery [34] (Figure 5).

Notably, recent work has shown that NBR1 can also recognize non-ubiquitinated substrates through liquid–liquid phase separation (LLPS). The ZZ and FW domains of NBR1 mediate phase separation prior to their autophagic degradation, suggesting that condensate formation serves as a prelude to cargo recognition and delivery to the vacuole [58]. ERC1 (ELKS/Rab6-interacting/CAST1) proteins can coalesce with NBR1 and ATG8e into membraneless condensates, thereby facilitating phase separation-dependent recruitment of NBR1 to the autophagic machinery [13] (Figure 5).

NBR1-mediated selective autophagy also influences plant heat responses by modulating the stability of key regulatory transcriptional factor proteins. In tomato, WRKY33 is required for proper heat responses, and its silencing compromises heat tolerance while reducing heat-induced *ATGs*’ expression and autophagosome formation [59] (Figure 5). In parallel, NBR1 affects thermotolerance through the turnover of HSP90 and ROF1 under heat stress. NBR1 promotes the degradation of these proteins, whereas NBR1 deficiency leads to their stabilization, enhanced *HSFA2* expression and transcriptional activity, and the subsequent induction of *HSP* genes, ultimately improving plant heat tolerance [60] (Figure 5).

### 4.2. NBR1-Mediated Selective Autophagy in Plant Chilling Tolerance

Similar to heat and drought stress, chilling stress also imposes a severe challenge to cellular proteostasis. Reduced enzymatic activity, decreased membrane fluidity, and compromised protein folding under low temperature collectively promote the accumulation of misfolded and aggregation-prone proteins. The efficient removal of these cold-induced aggregates is therefore critical for cellular homeostasis and plant survival. Given the limited capacity of the UPS to clear aggregated proteins [61], NBR1-dependent selective autophagy provides an important complementary pathway for their recognition and removal, thereby supporting plant adaptation to low-temperature stress.

**Figure 6:**
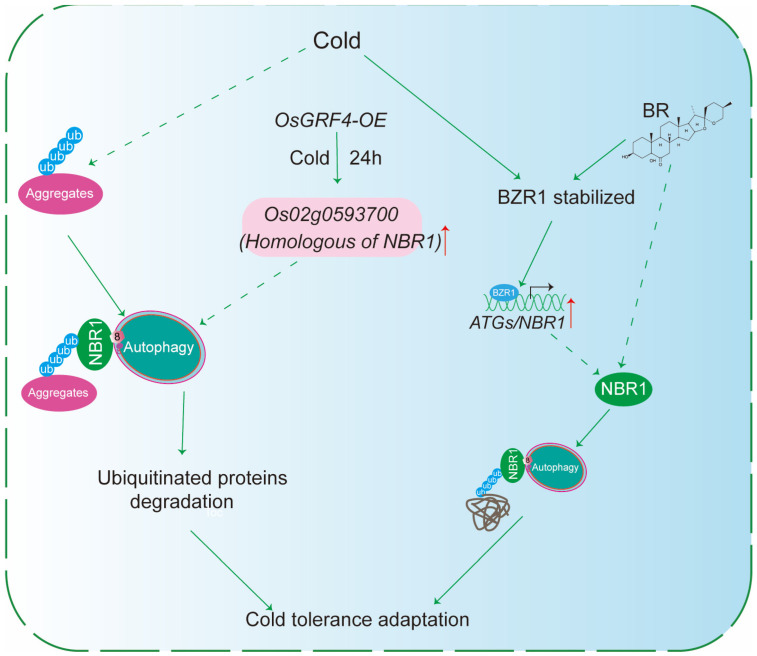
NBR1-mediated Selective Autophagy in Cold Stress Tolerance. Under cold stress *OsGRF4-*overexpressing lines (*OsGRF4-OE*) are exposed to cold for 24 h. Cold stress induces the formation of protein aggregates that are heavily ubiquitinated. The rice protein Os02g0593700, which is a homolog of the autophagy cargo receptor NBR1, is recruited to these ubiquitinated aggregates. NBR1 then engages the autophagy machinery. Through NBR1-mediated selective autophagy, ubiquitinated proteins are degraded. As a consequence of autophagic clearance, the transcription factor BZR1 is stabilized. Finally, both BZR1 and the core autophagic components (ATGs/NBR1) contribute to cold tolerance adaptation. Solid arrows in the diagram represent experimentally supported steps (e.g., NBR1 binding to ubiquitinated aggregates, autophagic degradation, and BZR1 stabilization), whereas the relationship between OsGRF4 overexpression and the induction/activity of the NBR1 homolog is currently proposed (indicated by dashed arrows in the diagram).

In addition to the direct elimination of protein aggregates, this pathway is also shaped by upstream regulatory networks. Notably, Brassinosteroids (BRs) have emerged as important regulators of plant tolerance to low-temperature stress. BR signaling contributes to the maintenance of cellular proteostasis under adverse conditions, suggesting a functional link between BR-dependent regulatory networks and NBR1-dependent selective autophagy pathways responsible for aggregate clearance during chilling stress. In tomato, both BR application and cold exposure stabilize the BR-responsive transcription factor BZR1, which subsequently activates the transcription of several *ATGs*, including *ATG2*, *ATG6*, and *SINBR1a/b* (Figure 6). Elevated *NBR1* levels, in turn, promote the selective turnover of defective and aggregation-prone proteins that are marked by extensive ubiquitination, thereby enhancing chilling tolerance [62].

Silencing *NBR1* or core *ATGs* compromises BR-induced cold tolerance, resulting in increased accumulation of ubiquitinated proteins and reduced levels of functional proteins (Figure 6). Together, these findings support a model in which BRs, through BZR1, activate NBR1-dependent selective autophagy to clear chilling-induced protein aggregates and preserve cellular function at low temperatures [62]. Beyond BR-dependent signaling, additional transcriptional regulators may also modulate NBR1-related proteostasis pathways during cold stress. For example, Growth-regulating Factor4 (GRF4) has been identified as a key transcription factor that promotes the expression of *LOC_Os02g38050*, an NBR1 homolog in *Oryza sativa*, under cold stress (Figure 6). Consistent with this, RNA-seq analysis of *OsGRF4*-overexpressing lines showed increased expression of *LOC_Os02g38050* together with reduced accumulation of ubiquitinated proteins relative to wild-type plants [63] (Figure 6).

### 4.3. NBR1-Mediated Selective Autophagy in Plant Salt Tolerance

**Figure 7:**
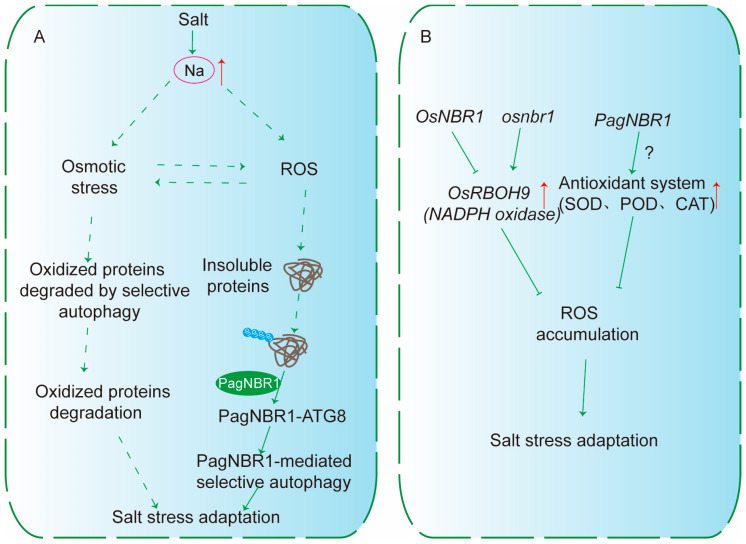
NBR1-mediated Selective Autophagy in Plant Salt Stress Adaptation. (**A**) Salt stress simultaneously triggers osmotic stress, Na+ accumulation, and ROS production. These stress signals lead to protein oxidation and the formation of insoluble proteins. Such damaged proteins are degraded via selective autophagy. In poplar, PagNBR1 interacts with ATG8 to initiate PagNBR1-mediated selective autophagy. (**B**) NBR1 regulates ROS homeostasis and salt stress adaptation in rice and poplar. In rice, OsNBR1 is shown in relation to *OsRBOH9* (a NADPH oxidase). The OsNBR1 negatively regulates ROS levels, as *Osnbr1* mutants display increased ROS accumulation. NBR1 in poplar can also activate the antioxidant system (e.g., SOD, POD, CAT), leading to reduced ROS accumulation and ultimately enhancing salt stress adaptation. Solid arrows indicate experimentally supported connections. Dashed arrows represent proposed mechanisms. The ‘?’ in (**B**) indicates that the mechanism by which PagNBR1 regulates the antioxidant system remains unknown.

Salt stress induces osmotic, ionic, and oxidative disturbances, including ROS accumulation, which impair plant growth. In response, plants activate autophagy to recycle nutrients, regulate osmotic balance, and facilitate stress adaptation (Figure 7A).

Beyond its functions under temperature-related stresses, NBR1-mediated selective autophagy also contributes to plant adaptation to salinity. Salt stress triggers a complex array of physiological disturbances, including osmotic, ionic, and oxidative stresses, which collectively attenuate plant growth. In response, plants activate the autophagy system to recycle nutrients, regulate osmotic balance, and facilitate Na+ redistribution, thereby mitigating stress damage.

Independent studies across different species have demonstrated that NBR1-mediated selective autophagy plays an important role in salt stress response. For example, in *Arabidopsis*, autophagy is rapidly activated within as little as 30 min of salt exposure, as evidenced by the accumulation of autophagosomal vesicles in root cells and increased levels of NBR1, which are associated with autophagic activity [64]. In Populus, salt stress induces the expression of *PagNBR1*, which interacts with PagATG8 to initiate selective autophagy (Figure 7A). This process enhances antioxidant enzymatic capacity, facilitates ROS detoxification, and promotes the clearance of both non-ubiquitinated substrates and insoluble ubiquitinated protein aggregates generated under saline conditions. Specifically, the antioxidant enzymes such as SOD, POD, and CAT exhibit no difference under normal conditions; however, the activities of all of them are dramatically upregulated when treated with salt for 4 days [65] (Figure 7B).

Similarly, in *Oryza sativa*, the autophagy receptor OsNBR1 regulates salt stress tolerance by modulating ROS homeostasis. Overexpressing *OsNBR1* accelerates ROS clearance and enhances salt tolerance, whereas *osnbr1* mutant exhibits excessive ROS accumulation, accompanied by upregulation of *OsRBOH9* (respiratory burst oxidase homolog) [66] (Figure 7B).

### 4.4. NBR1-Mediated Selective Autophagy in Metal Tolerance

NBR1-mediated selective autophagy serves as a central mechanism for maintaining proteostasis across a wide range of environmental challenges. Following its established roles in temperature and salinity stress, recent studies have highlighted its importance in mitigating the deleterious effects of heavy metal toxicity, including ROS accumulation, lipid peroxidation, and oxidative damage.

**Figure 8:**
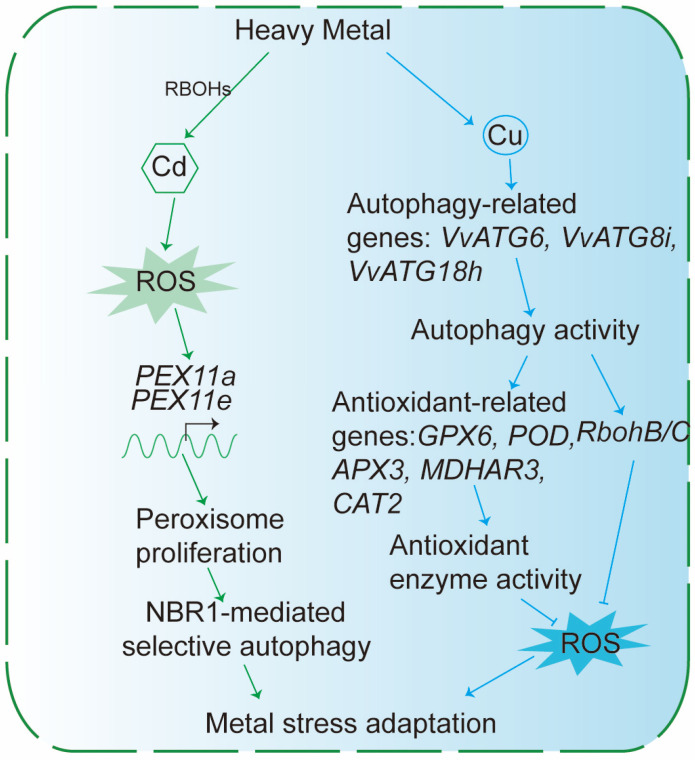
Role of NBR1-mediated Selective Autophagy in Plant Tolerance to Heavy Metals. Exposure to heavy metals such as Cd and Cu induces ROS production via RBOHs. Under Cd stress, ROS activates peroxisome proliferation by inducing the expression of *PEX11a* and *PEX11e*. Together with NBR1-mediated selective autophagy, damaged peroxisomes are removed, and ROS-dependent gene expression is regulated, ultimately contributing to metal stress adaptation. Under Cu stress, the upregulation of autophagy-related genes (e.g., *VvATG6*, *VvATG8i*, and *VvATG18h*), leading to increased autophagy activity. This in turn enhances the expression of antioxidant-related genes (e.g., *GPX6*, *POD*, *APX3*, *MDHAR3*, and *CAT2*) while downregulating RbohB/C. The combined effect is increased antioxidant enzyme activity, reduced ROS levels, and improved Cu tolerance.

Specifically, heavy metal exposure impairs plant growth and cellular homeostasis through multiple mechanisms. Excessive heavy metals compromise antioxidant defense systems and exacerbate oxidative stress, as reflected by elevated levels of ROS, malondialdehyde (MDA), and hydrogen peroxide (Figure 8). ROS production is tightly regulated by a network of factors, including members of the NADPH oxidase RBOH family [67], and glycolate oxidase (GOX) [68,69]. These ROS-regulating factors collectively influence pexophagy, a selective form of autophagy mediated by receptors such as NBR1, thereby linking heavy metal-induced oxidative stress to NBR1-dependent proteostasis mechanisms.

Among heavy metals, copper (Cu) exemplifies the dual role of essential micronutrients and potential toxicants. Cu is required for photosynthesis, transpiration, stomatal regulation, and antioxidant defense. Yet excess Cu promotes ROS production and oxidative damage. In *Vitis vinifera*, Cu stress induces the upregulation of key autophagy-related genes, including *VvATG6*, *VvATG8i*, and *VvATG18h*, enhancing ROS clearance through autophagy by activating the expression of antioxidant-related genes, such as, *GPX6*, *POD*, *APX3*, *MDHAR3*, and *CAT2*, while concurrently downregulating *RbohB* and *RbohC*, two genes involved in ROS production [70,71] (Figure 8).

By contrast, Cu deficiency impairs photosynthetic capacity and perturbs mitochondrial electron transport, concomitantly reducing Cu/Zn superoxide dismutase activity and leading to ROS accumulation and oxidative damage [72].

Recent studies have extended these findings to cadmium (Cd). Cd exposure triggers ROS accumulation, activates the unfolded protein response (UPR), and compromises antioxidant capacity demonstrating that this heavy metal also activates ROS accumulation and engages NBR1-dependent selective autophagy to maintain cellular homeostasis [73,74,75]. In addition to the upregulation of core autophagy components, Cd stress induces elevated expression of the autophagy receptor NBR1. In *Arabidopsis*, Cd exposure promotes transient peroxisome proliferation coupled with activation of the autophagy machinery, as indicated by the dynamics of molecular markers including ATG8 and PEX14a (Figure 8). High-resolution subcellular localization further demonstrates colocalization of AtATG8a and AtNBR1 within the peroxisomal matrix, suggesting that NBR1 selectively mediates autophagic turnover of peroxisomes [76]. More recent studies have shown that both NBR1 and HIPP33 (heavy metal-associated isoprenylated plant protein33) function as selective autophagy receptors to mediate the vacuolar sequestration of Cd, highlighting the versatile role of NBR1 in coordinating heavy metal detoxification through selective autophagy [77,78].

### 4.5. Interactions Between Plant Hormones and NBR1-Mediated Selective Autophagy in Abiotic Stress Responses

While NBR1-mediated selective autophagy mitigates the proteotoxic effects of various abiotic stresses, increasing evidence indicates that its activity is further regulated by plant hormone signaling networks, providing an additional layer of control over stress adaptation. Plant hormones such as ABA, SA, and BRs are essential for coordinating growth, development, and stress responses, and studies suggest that these phytohormones can modulate NBR1-mediated selective autophagy to fine-tune plant adaptation under environmental stress (Table 1).

For example, BR signaling positively regulates NBR1-mediated selective autophagy during chilling stress in tomato. The BR-responsive transcription factor BZR1 activates the transcription of NBR1, thereby enhancing selective protein degradation and improving cold tolerance [62] (Figure 6). Similarly, SA signaling modulates autophagy in *Arabidopsis*: elevated SA levels or overexpression of the SA regulator *NPR1* inhibit carbon starvation-induced autophagy and accelerate leaf senescence, whereas mutation of *NPR1* enhances autophagosome formation and promotes autophagic degradation of NBR1 [54] (Figure 4C). These findings suggest that SA promotes carbon starvation-induced leaf senescence by negatively regulating selective autophagy through NPR1.

ABA signaling is also linked to NBR1 function in stress adaptation. Transcriptomic analyses of *AtNBR1*-overexpressing *Arabidopsis* lines revealed widespread changes in stress-responsive genes, with network analysis indicating strong connections to ABA signaling pathways [84]. Although endogenous ABA levels were not significantly altered, *AtNBR1*-overexpressing plants exhibited delayed seed germination and increased stomatal closure, whereas *NBR1* knockout mutants showed enhanced lateral root initiation. Furthermore, NBR1 interacts with key ABA regulators, including ABI3, ABI4, and ABI5, suggesting that it may fine-tune ABA signaling by modulating the stability or activity of these proteins [84]. Together, these studies indicate that NBR1 coordinates selective autophagy with hormone-mediated stress responses in plants.

## 5. NBR1-Dependent Selective Autophagy in Biotic Stresses

Selective autophagy plays a crucial role in plant responses to biotic stresses, serving as a key regulatory mechanism that modulates the plant immune system. In the face of pathogen attacks, autophagy facilitates the selective degradation of damaged or misfolded proteins, as well as the turnover of intracellular pathogens, thus contributing to both plant defense and survival. Importantly, selective autophagy is intimately linked with the plant’s ability to interact with various pathogens, including bacteria and viruses, where it can be hijacked by virulent effectors to subvert host defense mechanisms. In turn, bacteria and viruses have evolved strategies to form granules of NBR1 to escape degradation, further illustrating how pathogens manipulate host autophagic pathways for their own benefit. In particular, the NBR1-dependent selective autophagy pathway has emerged as a significant player in mediating the plant’s defense against biotic stressors, setting the stage for exploring its interactions with bacterial and viral effectors, as well as the interplay between the UPS and autophagic pathways.

### 5.1. NBR1-Mediated Selective Autophagy in Bacterial and Fungal Infections

Bacterial effectors have evolved complex mechanisms to manipulate host autophagy, modulating the process to either promote infection or suppress plant immune responses. By directly or indirectly targeting key autophagy components and catabolic factors, these effectors facilitate bacterial proliferation and disease progression, ultimately enabling the pathogen to overcome host defenses [85].

**Figure 9:**
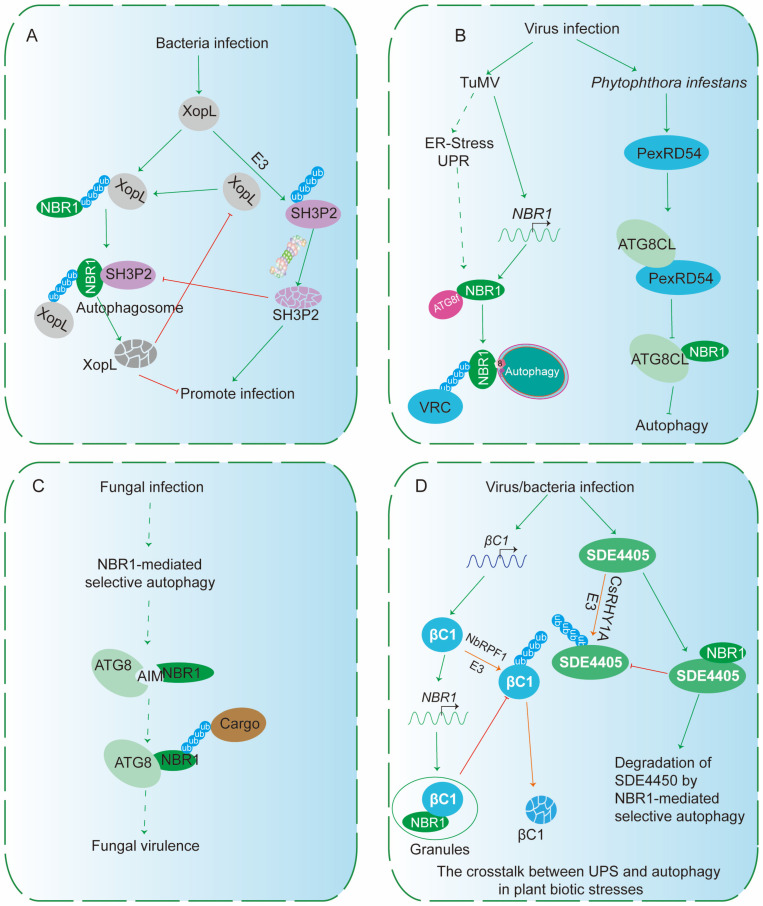
NBR1-mediated Autophagy in Plant Biotic Stresses. This figure presents a working model for the roles of NBR1-mediated autophagy during bacterial, viral, oomycete, and fungal infections, as well as the crosstalk between autophagy and the UPS. (**A**) Bacterial infection. Bacteria secrete the effector protein XopL, which functions as a ubiquitin E3 ligase and suppresses autophagy by targeting SH3P2 (an autophagy adaptor) for degradation via the UPS. However, NBR1 can recognize and target XopL for autophagic degradation, suggesting that XopL may subvert autophagy for pathogen benefit. (**B**) TuMV infection induces ER stress and the UPR, which activates NBR1-dependent autophagy by inducing the expression of *NBR1*; this autophagic process targets the TuMV NIb, the RNA-dependent RNA polymerase of the viral replication complex (VRC) to the tonoplast, promoting viral replication and virion accumulation. During oomycete infection by *Phytophthora infestans*, the effector PexRD54 disrupts the interaction between the NBR1 and ATG8CL, competitively displacing NBR1 from ATG8CL. (**C**) Fungal infection. Fungal infection activates NBR1-mediated selective autophagy. NBR1 contains an AIM that binds ATG8, to regulate fungal virulence. (**D**) Crosstalk between UPS and autophagy in plant biotic stresses. In *Citrus sinensis*, SDE4405 is ubiquitinated by the E3 ligase CsRHY1A and targeted for proteasomal degradation, but its interaction with CsNBR1 prevents this degradation. During geminivirus infection, the βC1 protein is induced, and its accumulation upregulates *NbNBR1* expression, leading to cytoplasmic granules where NbNBR1 interacts with βC1, thereby protecting βC1 from UPS-mediated degradation by NbRFP1 and enhancing viral accumulation. Solid arrows indicate experimentally supported connections. Dashed arrows represent proposed mechanisms.

The effector protein XopL, secreted by *Xanthomonas campestris pv. Vesicatoria* (Xcv), possesses E3 ubiquitin ligase activity, which it utilizes to suppress host autophagy [79] (Figure 9A). It achieves this by interacting with SH3P2, a crucial autophagy component involved in autophagosome formation, and promoting its proteasomal degradation [79]. Interestingly, NBR1/Joka2, an autophagy receptor, recognizes and directs XopL for autophagic degradation, illustrating a defensive response by the plant [79] (Figure 9A). This dynamic highlights a sophisticated arms race: while pathogens manipulate autophagy to undermine host immunity, plants employ their autophagic machinery to counteract bacterial effectors and attenuate effector-driven virulence, showcasing the intricate interplay between host defense and pathogen strategies.

Two effectors, SDE3 and SDE4405, from *Candidatus Liberibacter asiaticus* (CLas), the pathogen responsible for citrus Huanglongbing (HLB), have recently been shown to manipulate host autophagy in distinct ways [86,87]. While SDE3 targets citrus cytosolic glyceraldehyde-3-phosphate dehydrogenases (CsGAPCs), promoting their degradation of CsATG8 proteins and effectively inhibiting autophagic processes, SDE4405 takes a different approach by activating selective autophagy (Figure 9D). This is achieved through its direct interaction with both ATG8 and NBR1, facilitating the pathogen’s ability to hijack host autophagic machinery and enhance virulence [80].

Emerging evidence indicates that NBR1-mediated selective autophagy also plays pivotal roles in fungal development and pathogenicity. In the necrotrophic fungus *Sclerotinia sclerotiorum*, the core autophagy component SsATG8 and the selective cargo receptor SsNBR1 are indispensable for multiple aspects of fungal biology, including vegetative growth, sclerotial formation, oxalic acid production, compound appressoria development, and virulence [88]. Functional analyses further reveal that canonical AIM and UBA are required for these processes, underscoring the importance of selective cargo recognition in fungal autophagy [88] (Figure 9C). Notably, mutants lacking SsATG8 or SsNBR1 exhibit heightened sensitivity to proteasome inhibition and altered *ATGs*’ expression under proteotoxic stress, indicating a coordinated interplay between the UPS and NBR1-dependent selective autophagy. Together, these findings highlight that NBR1-dependent autophagy is not only integral to fungal morphogenesis and infection but also contributes to proteostasis through crosstalk with proteasomal degradation pathways.

Furthermore, autophagy in plants is crucial for regulating hormone levels and signaling, playing an essential role in basal resistance against hemibiotrophic bacteria and necrotrophic fungi. It also regulates defense-related cell death and contributes to processes linked to disease resistance [89]. Recent studies have further emphasized the importance of selective autophagy in plant immune responses, such as the discovery of an ATG8-binding oomycete effector that disrupts the interaction between the autophagic cargo receptor NBR1 [90,91]. This finding highlights the central role of autophagic regulation in plant defense mechanisms and suggests that pathogens may target this pathway to subvert host immune responses.

### 5.2. NBR1-Mediated Selective Autophagy in Viral Restriction

Autophagy serves as a crucial antiviral defense mechanism in plant–virus interactions, targeting several viral virulence factors for degradation through NBR1-dependent selective autophagy. For instance, the βC1 protein of *Cotton leaf curl Multan virus* (CLCuMuV) directly interacts with ATG8, leading to its autophagic degradation [81]. Additionally, in tomato, studies reveal that the βC1 protein further binds to the autophagic receptor NbNBR1, forming cytoplasmic granules [82] (Figure 9D). This interaction protects βC1 from UPS-mediated proteolysis, demonstrating the virus’s strategy to manipulate both autophagic and proteasomal pathways to enhance infection (Figure 9D).

*Turnip mosaic virus* (TuMV), a positive-stranded RNA potyvirus, can be suppressed by NBR1-mediated selective autophagy [92]. This process targets the viral RNA silencing suppressor, helper-component proteinase (HCpro), likely in association with virus-induced RNA granules [92]. Through this mechanism, the host utilizes autophagy to limit the virus’s ability to suppress RNA silencing, thereby enhancing antiviral defense.

Similarly, TuMV activates UPR-dependent NBR1-ATG8f autophagy to target the virus replication complex (VRC) to the tonoplast, promoting viral replication and virion accumulation [93] (Figure 9B). In a similar vein, *tombusviruses*, such as *Tomato bushy stunt virus* (TBSV), hijack selective autophagy by recruiting key autophagy proteins like ATG8f and NBR1 to viral replication organelles (VROs), facilitating viral replication [83]. In both cases, the host utilizes autophagy to regulate viral infection, either by limiting viral suppression of RNA silencing or by enhancing viral replication through the formation of specialized membrane structures.

In the case of the DNA virus, *cauliflower mosaic virus* (CaMV), the autophagy cargo receptor NBR1 targets unassembled capsid proteins and those involved in virus particle formation, facilitating their NBR1-dependent selective autophagic degradation [94]. This process plays a crucial role in restricting the establishment of CaMV infection, highlighting the importance of NBR1-mediated selective autophagy in plant antiviral defense.

### 5.3. Pathogen Manipulation of Autophagy in Oomycete Infections

Oomycete pathogens, such as *Phytophthora infestans*, have evolved effector proteins to manipulate autophagy for immune evasion, nutrient acquisition, and maintenance of biotrophy. For example, the effector PexRD54 reprograms host selective autophagy by disrupting the interaction between the antimicrobial receptor Joka2/NBR1 and ATG8CL, thereby competitively displacing NBR1 from ATG8CL and redirecting autophagic flux [95] (Figure 9B). Notably, defense-associated ATG8CL/Joka2-labeled autophagosomes are normally mobilized toward the pathogen interface to restrict pathogen proliferation, whereas PexRD54 relocalizes to these autophagosomes and co-opts them at the host–pathogen interface, a site that also serves as a hub for autophagosome biogenesis. This reprogramming underscores how *P. infestans* remodels host autophagy to attenuate immune responses and facilitate infection [95,96].

### 5.4. The Interplay Between NBR1-Mediated Selective Autophagy and UPS in Biotic Stresses

The cooperation between the UPS and autophagy is central to regulating plant immunity. Both pathways work together to balance the degradation of immune-related proteins and the selective turnover of cellular components, ensuring a robust response to microbial threats. UPS-mediated proteolysis enables the rapid modulation of immune receptor levels, while autophagy targets damaged or surplus proteins for degradation. Together, these processes optimize immune signaling and maintain cellular homeostasis, which is critical for plant defense against infections.

Proteasomal degradation, a tightly regulated process, plays a crucial role in immune responses and host–pathogen interactions across biological kingdoms [97,98,99,100,101,102]. Similarly, autophagy has emerged as a central process in immunity, particularly influencing plant health and responses to biotic stressors [39,103,104,105,106]. This underscores the crosstalk between UPS and autophagic pathways in immune regulation, and highlights how pathogens exploit these systems to promote infection.

For instance, in *Nicotiana benthamiana* infected with geminivirus, the βC1 protein encoded by the tomato yellow leaf curl China betasatellite (TYLCCNB) upregulates the expression of the autophagic receptor NbNBR1 [82] (Figure 9D). This leads to the formation of cytoplasmic granules, where NbNBR1 interacts with βC1, preventing it from degradation by the E3 ligase NbRFP1 and enhancing viral accumulation [82] (Figure 9D). This demonstrates how a viral satellite protein exploits the host’s autophagic machinery to protect itself from degradation, thereby enhancing viral accumulation and facilitating infection.

In *Citrus sinensis*, the effector protein SDE4405 is ubiquitinated by the E3 ligase CsRHY1A, targeting it for proteasomal degradation [80] (Figure 9D). However, SDE4405 interacts with the autophagic receptor CsNBR1, preventing its degradation by CsRHY1A and inhibiting CsNBR1-mediated autophagic degradation of another CLas effector, SDE1 [80]. This manipulation of both UPS and autophagic pathways by CLas further demonstrates the complexity of bacterial strategies in promoting infection and highlights the coordinated interaction between these two degradation systems in regulating plant immunity.

## 6. Conclusions and Future Perspectives

NBR1-mediated selective autophagy is emerging as a central regulator of plant proteostasis, development, and stress adaptation. By linking ubiquitinated or damaged proteins and organelles to ATG8-labeled autophagosomes, NBR1 ensures the selective clearance of cytotoxic aggregates, supports proper organelle turnover, and maintains cellular homeostasis under diverse abiotic and biotic stresses. Beyond its cargo recognition function, NBR1 integrates signals from phytohormones such as ABA, SA, and BRs [54,62,84], and coordinates with the UPS, highlighting its role as a hub connecting protein degradation, signaling networks, and stress resilience.

Despite these advances, key questions remain. The molecular mechanisms governing hormone-mediated regulation of NBR1, particularly under heat, drought, salinity, and cold stresses, are still largely unexplored. Similarly, the precise substrates and ubiquitin-like conjugation pathways targeted by NBR1 under specific stress conditions remain to be identified. Emerging proximity labeling technologies, such as TurboID, offer unprecedented opportunities to map NBR1 interactomes and cargoes in vivo [107,108,109,110,111], potentially revealing how selective autophagy dynamically adjusts proteostasis in response to environmental cues. For example, studies on Golgi recovery under heat stress have shown that ATG8 translocates to swollen Golgi membranes, suggesting a potential link between NBR1 and selective autophagy in response to heat-induced cellular damage [111]. Analogously, in mammalian cells, TurboID labeling of the NBR1 homolog SQSTM1/p62 has enabled high-resolution mapping of the SQSTM1-associated proteome, successfully capturing aggregation-prone substrates and locally enriched stress-related factors [107]. These findings demonstrate that TurboID can robustly identify selective autophagy cargoes within their native cellular context, highlighting its potential for elucidating NBR1 substrate dynamics in plants.

Future studies combining genetic, biochemical, and high-resolution proteomic approaches will be essential to fully elucidate NBR1′s regulatory network. Understanding the interplay between NBR1, hormone signaling, and the UPS will not only advance fundamental knowledge of plant selective autophagy but may also inform strategies to engineer crops with enhanced stress tolerance and developmental robustness.

## Figures and Tables

**Table 1:** Summary list of proteins interacting with NBR1 and/or ATG8 under plant stress conditions.

Species	Stress Context	Cargo	Interaction with ATG8 and/or NBR1	Evidence(s)	Reference(s)
Tomato	Heat	FL2SP	√	Proteolytic cleavage assay and co-localization	[55]
*Arabidopsis*	Drought	COST1	√	Co-localization, Luciferase analysis, and Pull-down	[56]
*Arabidopsis*	Light	Toc132	√	Co-localization	[10]
*Arabidopsis*	Heat	Toc33	√	Co-localization, BiFC, and Co-IP	[57]
*Arabidopsis*	Heat	Toc75	√	Co-localization, BiFC, and Co-IP	[57]
*Arabidopsis*	Heat	Toc159	√	Co-localization, BiFC, and Co-IP	[57]
*Arabidopsis*	Heat	ERC1	√	Co-localization, Pull-down, and Co-IP	[13]
Tomato	Heat	HSP90	√	Co-localization and Co-IP	[60]
Tomato	Heat	ROF1	√	Co-localization and Co-IP	[60]
Tomato	Cold	BZR1	√	Y1H and ChIP-qPCR	[62]
Poplar	Salt	N/A	×	×	[65]
Rice	Salt	N/A	×	×	[66]
*Arabidopsis*	Cadmium	N/A	×	×	[76]
Xcv	Bacterial infection	XopL	√	Co-IP and co-localization	[79]
CLas	Bacterial infection	SDE4405	√	BiFC, Y2H, Co-IP, and Pull-down	[80]
CLCuMuV	Viral infection	βC1	√	Y2H, Pull-down, Co-IP, and BiFC	[81,82]
TBSV	Viral infection	TBSV p33	√	Co-localization, Pull-down, and Co-IP	[83]

Note: “N/A” indicates that no cargo substrate has been discovered; “√” indicates that a cargo protein has been discovered to interact with NBR1 and/or ATG8; “×”indicates that there is no interaction between the cargo protein and NBR1 and/or ATG8.

## Data Availability

The original contributions presented in the study are included in the article, further inquiries can be directed to the corresponding authors.

## Abbreviations

The following abbreviations are used in this manuscript:

ROS	Reactive Oxygen Species
NBR1	NEIGHBOR BRCA1 GENE1
BR	BRassinosteroids
ABA	Abscisic Acid
SA	Salicylic Acid
CMA	Chaperon-Mediated Autophagy
UBA	UBiquitin-Associated
AIM	ATG8-Interacting Motif
TOR	Target Of Rapamycin
PAS	Phagophore Assembly Site
ER	Endoplasmic Reticulum
ATG	AuTophaGy-related
PI3P	PhosphatidylInositol 3-Phosphate
PI3K	PhosphatidylInositol 3-Kinase
PE	PhosphatidylEthanolamine
SARs	Selective Autophagy Receptors
PB1	Phox and Bem1
ConA	Concanamycin A
ARF	Auxin Response Factor
PBS	PreBranch Site
HSP	Heat Shock Protein
FL2	FLOURY2
TOC	Translocon on the Outer Chloroplast membrane
LLPS	Liquid–Liquid Phase Separation
ERC1	ELKS/Rab-interacting/CAST1
UPS	Ubiquitin–Proteasome System
GRF4	Growth-Regulating Factor4
RBOH9	Respiratory Burst Oxidative Homologue9
MDA	MalonDiAldehyde
GOX	Glycolate OXidase
Cu	Copper
Cd	Cadmium
HIPP33	Heavy metal-associated Isoprenylated Plant Protein33
TuMV	Turnip Mosaic Virus
HCpro	Helper-Component proteinase
VRC	Virus Replication Complex
TBSV	Tomato Bushy Stunt Virus
VROs	Viral Replication Organelles
CaMV	Cauliflower Mosaic Virus
